# Multifunctional Oxidized Dextran Cross-Linked Alkylated Chitosan/Drug-Loaded and Silver-Doped Mesoporous Bioactive Glass Cryogel for Hemostasis of Noncompressible Wounds

**DOI:** 10.3390/gels9060455

**Published:** 2023-06-01

**Authors:** Dong Lei, Jing Zhao, Chenhui Zhu, Min Jiang, Pei Ma, Yu Mi, Daidi Fan

**Affiliations:** 1 Shaanxi Key Laboratory of Degradable Biomedical Materials, School of Chemical Engineering, Northwest University, Xi’an 710069, China; leidong1010@163.com (D.L.); zhaojing@nwu.edu.cn (J.Z.); zch2005@nwu.edu.cn (C.Z.); mapei@nwu.edu.cn (P.M.); 2 Shaanxi R&D Center of Biomaterials and Fermentation Engineering, School of Chemical Engineering, Northwest University, Xi’an 710069, China; 3 Biotech & Biomed Research Institute, Northwest University, Xi’an 710069, China; 4 State Key Laboratory of Materials-Oriented Chemical Engineering, College of Biotechnology and Pharmaceutical Engineering, Nanjing Tech University, Nanjing 211816, China; jiangmin@njtech.edu.cn

**Keywords:** shape-memory hemostatic cryogel, wound healing, coagulation disorders, noncompressible wounds

## Abstract

Noncompressible wounds resulting from accidents and gunshots are typically associated with excessive bleeding, slow wound healing, and bacterial infection. Shape-memory cryogel presents great potential in controlling the hemorrhaging of noncompressible wounds. In this research, a shape-memory cryogel was prepared using a Schiff base reaction between alkylated chitosan (AC) and oxidized dextran (ODex) and then incorporated with a drug-laden and silver-doped mesoporous bioactive glass (MBG). Hydrophobic alkyl chains enhanced the hemostatic and antimicrobial efficiency of the chitosan, forming blood clots in the anticoagulated condition, and expanding the application scenarios of chitosan-based hemostats. The silver-doped MBG activated the endogenous coagulation pathway by releasing Ca^2+^ and prevented infection through the release of Ag^+^. In addition, the proangiogenic desferrioxamine (DFO) in the mesopores of the MBG was released gradually to promote wound healing. We demonstrated that AC/ODex/Ag-MBG DFO(AOM) cryogels exhibited excellent blood absorption capability, facilitating rapid shape recovery. It provided a higher hemostatic capacity in normal and heparin-treated rat-liver perforation-wound models than gelatin sponges and gauze. The AOM gels simultaneously promoted infiltration, angiogenesis, and tissue integration of liver parenchymal cells. Furthermore, the composite cryogel exhibited antibacterial activity against Staphylococcus aureus and Escherichia coli. Thus, AOM gels show great promise for clinical translation in treating lethal, noncompressible bleeding and the promotion of wound healing.

## 1. Introduction

Uncontrolled bleeding presents a significant fatality risk in various settings, including battlefields, emergencies, and hospitals, contributing to over 5.8 million deaths worldwide annually [1,2]. Among these cases, noncompressible hemorrhage accounts for approximately 42% of trauma-related deaths [3]. Noncompressible hemorrhage commonly occurs in appendicular wounds, which are typically irregular or penetrating in nature, such as those caused by gunshots [4,5]. The conventional method for addressing bleeding in such wounds involves filling them with gauze [6]. However, due to the irregular and narrow nature of such wounds, gauze is often ineffective in stopping bleeding [7]. Other hemostatic agents, including hemostatic powder and gelatin sponges, have been developed for hemorrhage control, but they often prove ineffective for this type of wound [8,9,10,11,12,13]. Recently, shape-memory hemostats have shown promise in controlling noncompressible hemorrhage. However, existing devices such as the XstatTM are nonbiodegradable and require surgical removal following hemostasis [13,14]. Thus, developing biodegradable shape-memory hemostatic agents that do not require surgical removal would be advantageous. 

Chitosan (CS) is a naturally occurring polysaccharide with a positive charge, exhibiting hemostatic, nontoxic, antibacterial, and biodegradable properties [15,16,17]. The hemostatic ability of chitosan primarily arises from electrostatic interactions between protonated amino groups and negatively charged membranes of erythrocytes [18]. However, research has indicated that hemostats solely composed of CS have limited hemostatic efficiency and inferior mechanical strength in certain applications [19,20]. Alkylated chitosan (AC), a hydrophobic alkyl-chain-grafted CS, has been demonstrated to possess enhanced hemostatic and antimicrobial effects due to interactions between the hydrophobic alkyl chains and red blood cells, platelets, and bacterial membranes [21]. Unlike CS, AC was shown to form blood clots in anticoagulated conditions, expanding the application scenarios of CS-based hemostats to anticoagulated patients and patients with coagulation disorders [22]. Moreover, the hemostatic efficiency and mechanical strength can be further enhanced by developing CS-based composite cryogel hemostats. 

Bioactive ceramic materials, such as mesoporous bioactive glass (MBG), can absorb and concentrate blood components due to their mesoporous structures, thereby promoting wound hemostasis [23]. Recently, the US FDA approved a borate-based MBG for acute and chronic wound management (MIRRAGEN™, ETS Wound Care LLC, Rolla, MO, USA). This material absorbs wound fluids and creates a healing-enhancing environment for wounds [24]. MBG has been demonstrated to significantly enhance the mechanical strength and fluid absorption efficiency of CS-based hemostats [23]. MBG can be doped with ions to adjust its physical and biological properties. During severe bleeding, infection is a life-threatening yet frequently occurring event. MBG can be endowed with antimicrobial properties by being doped with Ag^+^, which is a broad-spectrum antibacterial agent. Recent research demonstrated that Ag^+^ has a lower possibility of causing bacterial resistance compared to Ag nanoparticles [25]. However, a major concern of antimicrobial Ag^+^ is its acute toxicity to normal tissue at high concentrations. The sustained release of Ag^+^ from ionically doped MBGs at appropriate levels during glass dissolution offers long-term antimicrobial effects with tolerable toxicity. Additionally, the released Ca^2+^ during MBG dissolution can activate the intrinsic coagulation cascade, thereby accelerating thrombin generation [26]. Consequently, incorporating silver-ion-doped MBG into the cryogel not only imparts antibacterial properties to the material but also enhances the hemostatic efficiency and improves the mechanical strength of the material. 

The aforementioned cryogel can accelerate the hemostatic and inflammatory phases of wound healing. Proangiogenic small-molecule Desferrioxamine (DFO) can be loaded into mesoporous MBG and released at the wound site to promote wound healing in the proliferation and remodeling phases. DFO promotes wound vascular regeneration by inducing the formation of hypoxia-inducible factor-1α (HIF-1α) [27]. However, its hydrophobic nature sets limitations on its application. The delivery of DFO employing MBG could address the solubility issue and facilitate sustained release for prolonged angiogenic effects [28].

Recently, shape-memory cryogels have been reported to effectively control noncompressible hemorrhaging due to highly interconnected and large porous structures, active coagulation, adaptability to wound shapes, and low cost [22,29]. However, the preparation of cryogels typically involves using highly reactive and toxic cross-linkers or initiators, which may lead to inflammation [30,31,32,33,34,35,36]. In previous research, we demonstrated that oxidized dextran (ODex) serves as a safe cross-linker for preparing CS-based cryogels [20].

In this research, composite hydrogels were prepared using ODex cross-linked alkylated chitosan (AC), which was incorporated with DFO-loaded and silver-doped mesoporous bioactive glass (MBG). The alkyl chains present in alkylated chitosan (AC) can insert into the blood-cell membrane and immediately form a clot with the blood and anchor the blood cells, thereby improving hemostatic efficiency. Importantly, the hemostatic mechanism of AC is independent of any part of the coagulation cascade, making it suitable for use in anticoagulant patients. Since the coagulation mechanism of AC does not depend on the coagulation cascade of the body, it can be applied to anticoagulant patients. The highly porous structure of Ag-doped MBG facilitates hemostasis, while the gradually released silver ions protect the wounds from inflammation. Furthermore, DFO can be continuously released from the mesopores of MBG to promote wound regeneration and thus promote wound healing.

## 2. Results and Discussion

### 2.1. Synthesis and Characterization of Cryogels

First, the synthesis and characterization of oxidized dextran (ODex), alkylated chitosan (AC), and silver-doped mesoporous bioactive glass (Ag-MBG) were performed. The successful oxidation of dextran was demonstrated through the FT-IR and 1H NMR spectra of dextran and oxidized dextran, as illustrated in Figure S1. The presence of characteristic peaks at 9.64 ppm and 9.20 ppm in the 1H NMR spectrum of oxidized dextran confirmed the presence of proton peaks corresponding to the aldehyde group. Additionally, the FT-IR spectrum of oxidized dextran exhibited an absorption peak at 1732 cm^−1^ corresponding to the aldehyde group. The degree of oxidation of ODex was determined to be 38.76% through hydroxylamine hydrochloride. Next, the successful synthesis of AC was confirmed using FT-IR and 1H NMR spectroscopy (Figure S2). AC was synthesized through a Schiff base reaction, followed by the conversion of the unstable C=N to the stable C-N using NaCNBH_3_. The FT-IR spectra demonstrated that AC shows characteristic peaks at 720 cm^−1^, 1650 cm^−1^, and 2800–3000 cm^−1^, indicating the successful reaction of amino and alkyl groups in CS. The 1H NMR spectra of AC exhibited characteristic peaks at 0.87 ppm and 1.27 ppm, corresponding to -CH_3_ and -CH_2_, both of which belong to the alkyl chain of AC. These findings show that the preparation of AC was successful. The degree of substitution of AC was determined to be 27.86 ± 18.99% through elemental analysis.

The morphology and particle size of MBG were examined using SEM and TEM. MBG particles are dispersed spheres with rough surfaces measuring approximately 200 nm in size, as illustrated in Figure S3. The TEM image reveals that Ag-MBG has a porous structure. As demonstrated in Figure S4, MBG exhibits a typical Type IV profile with a specific surface area of 502.24 m^2^ g^−1^, a total pore volume of 0.79 cm^3^, and an average pore size of 6.6 nm. As illustrated in Figure S5a, the bending vibrations at 461 cm^−1^ and stretching at 800 cm^−1^ and 1075 cm^−1^ show the amorphous structure of the silica network. This amorphous structure of MBG was further confirmed using XRD testing in Figure S4c. These findings demonstrate the successful synthesis of MBG. The MBG was doped with silver ions (Ag-MBG) to improve bacterial inhibition, while the mesopores were loaded with the proangiogenic molecule DFO to promote wound healing. As illustrated in Figure S5b, a peak belonging to Ag is observed at 44°, demonstrating the successful doping of silver ions. 

**Scheme 1:**
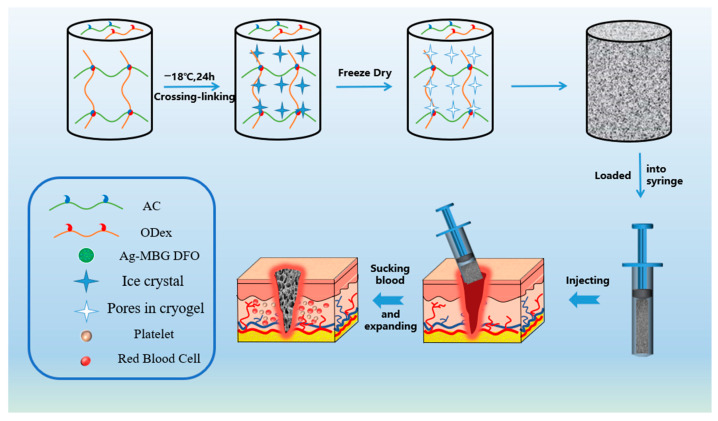
Schematic explanation of the preparation and application as cryogels.

The macroporous cryogels were prepared following the method shown in Scheme 1. Briefly, AC, ODex, and Ag-MBG DFO were homogeneously mixed in an ice bath and quickly brought to −20 °C for the cross-linking reaction. During this process, the water molecules in the system formed ice crystals and acted as porogenic agents, while the cryogel components became concentrated in the liquid microphase around the ice crystals [37]. Cryogels with varying concentrations of Ag-MBG DFOs (AO and AOM1-3) were synthesized, and the detailed formulations are presented in Table S1. The selection of the optimal cryogel formulation was conducted through single-factor optimization experiments, with the detailed information provided in the SI. Subsequently, the chemical structure of the cryogels was characterized using FT-IR spectroscopy. As illustrated in Figure S6, the AOM cryogels exhibited characteristic peaks corresponding to imine bonds at 1645 cm^−1^, while no characteristic peaks associated with aldehyde groups were observed, demonstrating that the aldehyde groups were fully reacted and the cryogels were chemically cross-linked through the Schiff base reaction between the amino group of AC and the aldehyde group of ODex.

Afterward, the loading and release of drugs from silver-doped mesoporous bioactive glasses were examined. As demonstrated in Table S2, the highest encapsulation and loading rates were achieved when the mass ratio of Ag-MBG to DFO reached 10/12. As illustrated in Figure S7, the drug was gradually released over 7 days and reached a plateau after day 7 when the accumulated release reached 50.18%. The higher drug release rate and long-term release could better promote wound healing.

**Figure 1:**
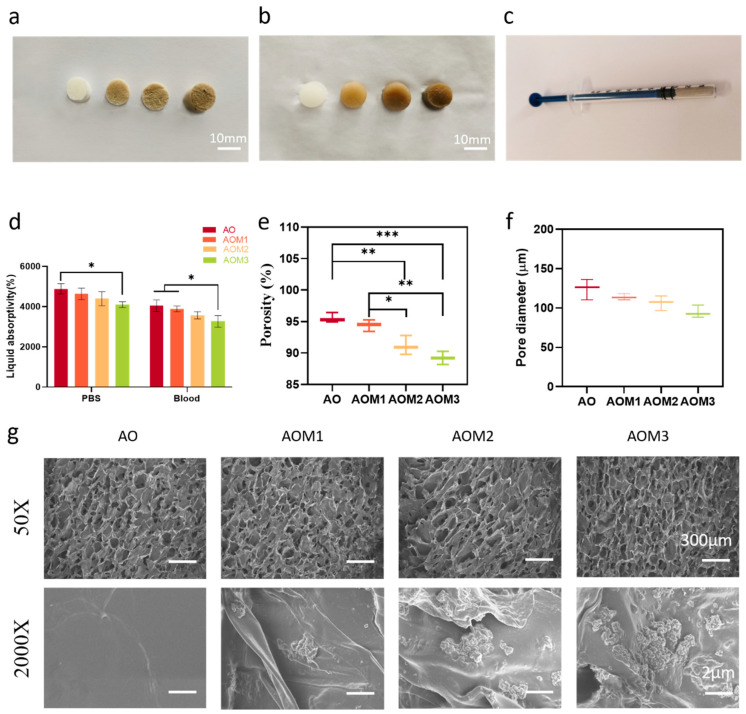
Characteristics of the cryogel. (**a**) Photograph of a freeze-dried cryogel; (**b**) Photograph of a cryogel in expansion equilibrium; (**c**) Photograph of the cryogel loaded into a syringe; (**d**) PBS/blood uptake rate of cryogels; (**e**) Porosity of cryogels; (**f**) Pore size distribution of cryogels; (**g**) SEM images of the surface morphology of different samples at different magnifications.

Figure 1a,b illustrate the color change of cryogel with the increase in Ag-MBG content, which changed from white to dark grey as the concentration of Ag-MBG changed from 0–0.6%. Figure 1c illustrates that the cryogel can be loaded into a syringe, showing the injectability of the cryogel.

Figure 1d shows the absorptivity of blood and PBS. When the cryogel reaches swelling equilibrium, it exhibits a high water/blood uptake rate, with the AO exhibiting the highest equilibrium ratio of 48.81. This high liquid absorption capacity is attributed to the interconnected macroporous structure, which physically traps water through capillary forces, as well as the hydrophilicity of the cryogel matrix. Compared to the cryogel’s uptake rate for PBS, its uptake performance for blood is slightly reduced, probably due to the high viscosity of blood. Figure 1d illustrates that an increase in MBG concentration leads to a decrease in the cryogel swelling ratio, possibly due to a reduction in the porosity and pore sizes of the MBG-incorporated cryogels (Figure 1e,f). Furthermore, the SEM images show the surface morphology of the cryogel in Figure 1g. All cryogels exhibit interconnected macroporous structures with pore sizes of approximately 100–200 µm. More Ag-MBG nanospheres could be observed on the wall of cryogel pores as the concentration of Ag-MBG increased from 0.2% to 0.6%.

The cryogel has high porosity, as illustrated in Figure 1e. As demonstrated in Figure 1f, the porosity of the four groups of cryogels was 95.54%, 94.38%, 91.15%, and 89.20%, and the mean pore sizes of AO, AOM1, AOM2, and AOM3 were 124.17 µm, 113.88 µm, 106.37 µm, and 94.77 µm, respectively. The decrease in pore sizes of the Ag-MBG-loaded cryogels may be attributed to the physical occupancy of the pores by the nanoparticles. The high liquid absorption and porosity of cryogels help them to rapidly absorb blood and aggregated blood cells, thereby accelerating hemostasis. In addition, the large pore structure of the cryogel absorbs exudate from the wound, thus reducing the chances of wound infection.

### 2.2. Mechanical and Shape Memory Properties of Cryogels

**Figure 2:**
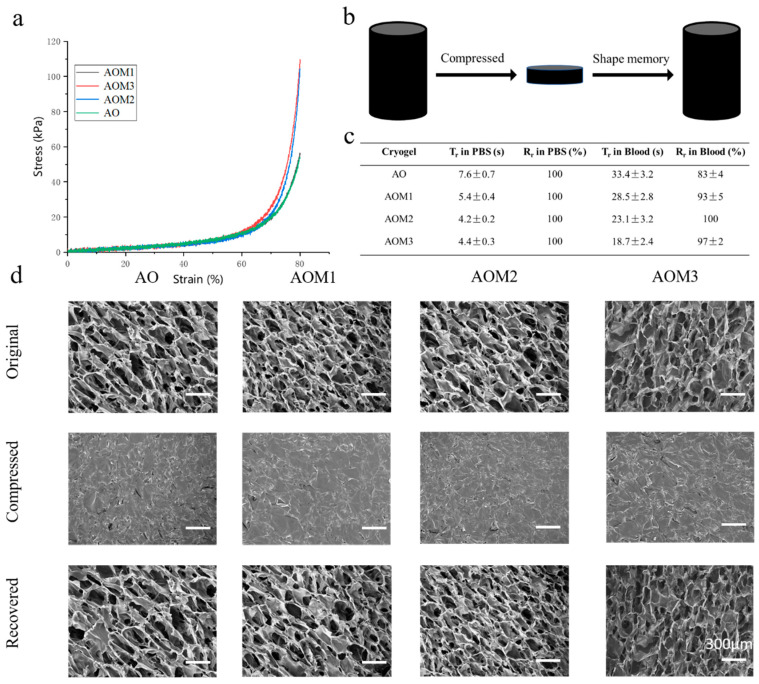
Mechanical performance and shape-memory properties of the cryogels. (**a**) Compression stress-strain curves of the cryogels; (**b**) Schematic diagram of the mechanism of shape memory performance of cryogels; (**c**) Shape recovery rate (Rr) and recovery time (Tr) in PBS/blood of the compressed cryogels; (**d**) SEM images of AO, AOM1, AOM2 and AOM3 in the original; compressed and recovered states. The scale bars in (**d**): 300 μm.

Cryogel is unique in its ability to control bleeding from noncompression wounds because of its shape memory ability, primarily due to the ability to absorb fluid and trigger shape recovery, leading to a physical blockage. The cryogel can be compressively loaded as in a syringe and injected into the wound site for hemostasis. Thus, the cryogel must possess excellent mechanical strength to be firmly plugged at the bleeding site. As illustrated in Figure 2a, the compression resistance of the cryogel increased from 53.44 kPa initially to 109.61 kPa as the concentration of Ag-MBG increased. This may be due to the doping of MBG, which dissipates the deformation energy of the material through physical occupation and enhances its mechanical strength. As demonstrated in Figure 2b, when the cryogel was compressed to 80%, causing water to flow out of its pores, it immediately absorbed water and expanded to its initial state upon pressure release. These experiments demonstrate that the cryogel exhibits good mechanical properties. As illustrated in Figure 2c, the cryogels exhibited better recovery rates in PBS and blood, with 100% shape recovery in PBS for all groups of gels, and the time required for recovery was less than 10 s. In particular, AOM2 required only 4.2 ± 0.2 s to recover to its initial state. The recovery time in blood follows a similar trend but is slightly longer than that in PBS because blood is more viscous. Figure 2d demonstrates that the compressed cryogel can almost fully recover to its initial volume after liquid absorption, displaying remarkable compression flexibility and shape memory. These findings demonstrate that the addition of Ag-MBG enhances the mechanical properties of the cryogel, and all formulations have the good shape-memory capability, attributed to their cross-linked and interconnected mesoporous structure.

### 2.3. Biocompatibility and Biodegradability of Cryogels

**Figure 3:**
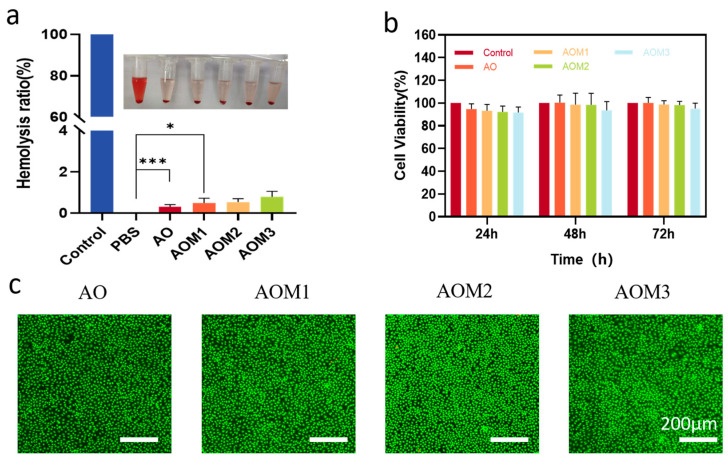
Biocompatibility of the cryogels. (**a**) Hemolysis ratio of the cryogels (from left to right:Control, PBS, AO, AOM1, AOM2, AOM3). (**b**) Cell viability of L929 cells incubated with the cryogel extracts for 1, 2, and 3 days. (**c**) AO/EB staining of L929 cells after incubating with the cryogel extracts for 1 day. The scale bars in (**c**): 200 μm.

When applied to the wound surface, cryogels must exhibit good biocompatibility. Hemolysis and MTT tests were conducted to assess their biocompatibility. Figure 3a illustrates the findings of the hemolysis test, indicating that the hemolysis rates of all cryogel groups were below 5%, demonstrating that the cryogel has excellent hemocompatibility. The quantitative findings showed that the hemolysis rates were less than 5% in all groups, demonstrating that the cryogel has good hemocompatibility. The cytotoxicity of cryogels was evaluated through MTT and AO/EB staining experiments. As illustrated in Figure 3b, all groups demonstrated good cellular activity >90% at all three time periods. However, the AOM3 group exhibited slightly lower cellular activity compared to the AOM2 group, possibly due to the high concentration of silver ions with slight toxicity. Furthermore, as demonstrated in Figure 3c, AO/EB staining showed the findings after 24 h of co-culture of the cryogels with L929 cells, with almost all cells in the cryogel group being stained green and showing little difference from the control group. These findings show that the cryogel can be safely employed for clinical applications.

**Figure 4:**
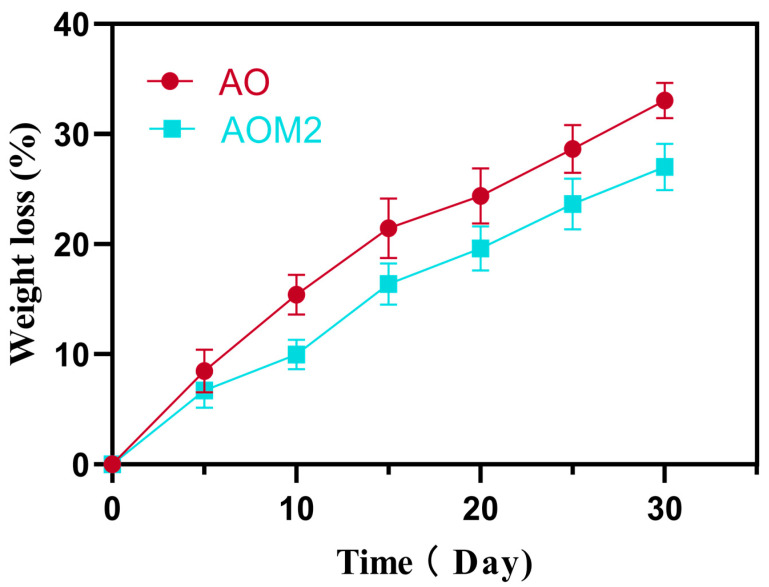
Degradability of the cryogels.

In clinical applications, the removal of hemostatic agents may result in secondary bleeding. Thus, an ideal hemostatic agent can be retained at the injury site and should be biodegradable. In vitro lysozyme degradation tests were conducted to assess the degradability of the cryogels and AO and AOM2 were selected for testing. Both cryogels demonstrated progressive degradation in vitro by lysozyme (Figure 4). After 30 days, AO achieved a degradation rate of 33.05%, while AOM2 exhibited a relatively slower in vitro degradation rate of 27.01%, probably due to the introduction of Ag-MBG leading to a slower degradation rate. These findings show that both cryogels are biodegradable and thus have great potential for in vivo application.

### 2.4. Antibacterial Properties of Cryogels

**Figure 5:**
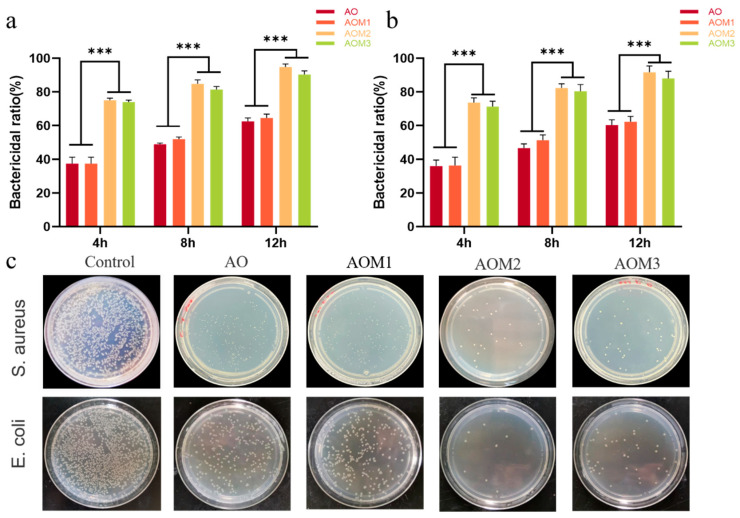
In vitro antibacterial activity of cryogels. (**a**,**b**) Bactericidal ratio of cryogels against *E. coli* and *S. aureus*.; (**c**) Photographs of colony-forming units of *E. coli* and *Staphylococcus aureus* growing on LB agar plates.

Bacterial infection poses a significant challenge to wound healing, and an ideal hemostasis dressing should also have anti-infective properties. Therefore, to evaluate the bactericidal rates of all cryogels, *E. coli* (Gram-negative bacteria) and *S. aureus* (Gram-positive bacteria) were employed. Figure 5a,b illustrate the bactericidal effect of cryogels. For antibacterial ability, AOM2 and AOM3 show a higher inhibitory effect than AO and AOM1, probably due to their higher concentration of silver ions. Notably, there was no significant difference between the antibacterial effects of AOM2 and AOM3. Among all tested cryogels, AOM2 was shown to be the most effective formulation, which led to over 85% bactericidal ratio at 12 h. Furthermore, Figure 5c presents photographs of colony-forming units of *E. coli* and *S. aureus* growing on LB agar plates. The AOM2 and AOM3 groups exhibited significantly fewer colony-forming units of bacteria compared to the other groups, which is consistent with the findings obtained from the antibacterial experiment. These findings show that the Ag-MBG-loaded cryogels have strong and prolonged antibacterial properties.

### 2.5. Hemostatic Properties of Cryogels

**Figure 6:**
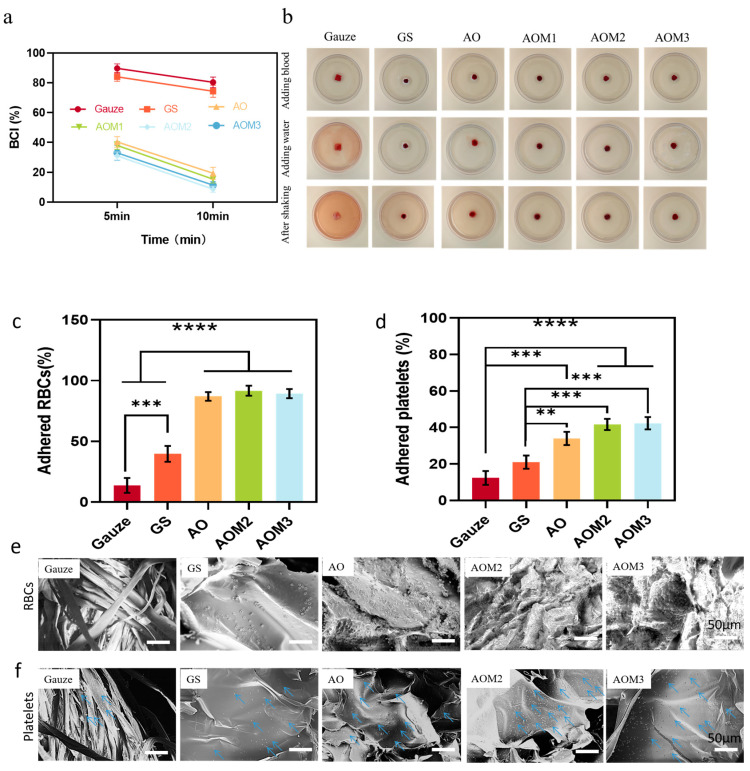
Hemostatic performance of cryogels in vitro. (**a**) BCI-time curves of the samples; (**b**) Photographs of the BCI assay process at 10 min; (**c**,**d**) Percentage of adhered RBCs and platelets on the samples; (**e**) SEM images of RBCs adhesion; (**f**) SEM images of platelets adhesion. The scale bars in (**e**,**f**): 50 μm.

The in vitro procoagulant properties of the cryogels were evaluated via whole blood coagulation assays using gelatin sponges (GS) and medical gauze as controls, where a lower blood coagulation index (BCI) value represented a greater procoagulant capacity of the cryogels. At 10 min, the BCI was 80.11%, 74.35%, 19.33%, 8.67%, and 11.12% for gauze, GS, AO, AOM1, AOM2, and AOM3, respectively (Figure 6a). Gauze exhibited the highest BCI, which was the worst procoagulant, followed by GS. The BCI was significantly lower in the cryogel group than in the control group at all time points tested in the experiment, indicating that the cryogel had a better procoagulant capacity. Among the cryogels, AOM2 indicated the lowest BCI and thus the highest hemostatic capacity. Furthermore, Figure 6b illustrates the findings of the whole blood coagulation test. After adding water to the GS and gauze groups, uncoagulated blood was released and exhibited a distinct red color when shaken in ultrapure water. However, there was almost no change in the cryogel group, which proves that the crystal gel has excellent procoagulant ability and can promote blood clotting on time. The findings demonstrate that AOM exhibits the best coagulation effect. Considering the effects of recovery time, BCI, and antimicrobial properties of the cryogels, we examined the adhesion and aggregation of RBCs and platelets on AO, AOM2, and AOM3. The impact of cryogels on the aggregation of blood cells and platelets was experimentally investigated. As illustrated in Figure 6c,d, the number of blood cells and platelets adsorbed and aggregated on the cryogel was significantly higher than that of the control group. SEM images revealed that as the concentration of Ag-MBG increased, significantly more erythrocytes and platelets were adsorbed onto the surface of the cryogel and maintained the distinctive biconcave disc shape (Figure 6e,f). The findings show that AOM2 and AOM3 have good procoagulant ability.

**Figure 7:**
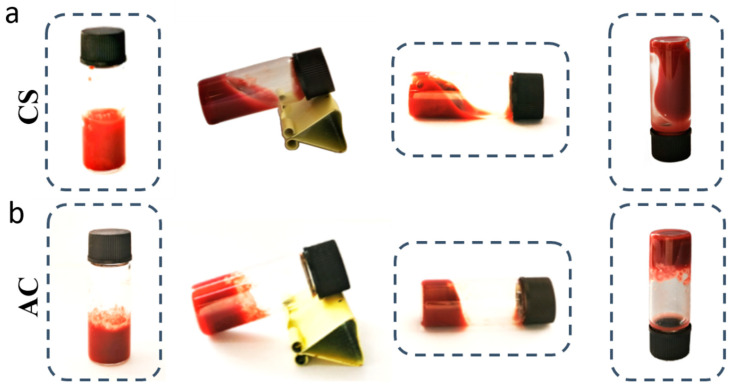
Coagulation performance of different materials against coagulation of whole blood.

Then, we examined the coagulation ability of chitosan and alkylated chitosan on anticoagulated whole blood, as illustrated in Figure 7, where a mixture of blood and chitosan/alkylated chitosan was added to sample bottles to assess the procoagulation capacity of chitosan/alkylated chitosan powders. By placing the sample bottles at an angle on the shelf, the mixture of chitosan powder and blood was in a liquid-flowing state, while the mixture of alkylated chitosan and blood was in a gel-like state. Subsequently, by placing the sample bottles at 90° flat and inverted, the sample with the addition of chitosan powder and blood was not solidified, whereas the mixture of alkylated chitosan powder and blood transformed into a gel. In the inversion experiments, the mixture of alkylated chitosan and blood could withstand its weight in the bottle, but the mixture of chitosan and blood flowed down the bottle walls. These findings indicate that alkylated chitosan possesses a greater procoagulant capacity for anticoagulated whole blood compared to chitosan. 

**Figure 8:**
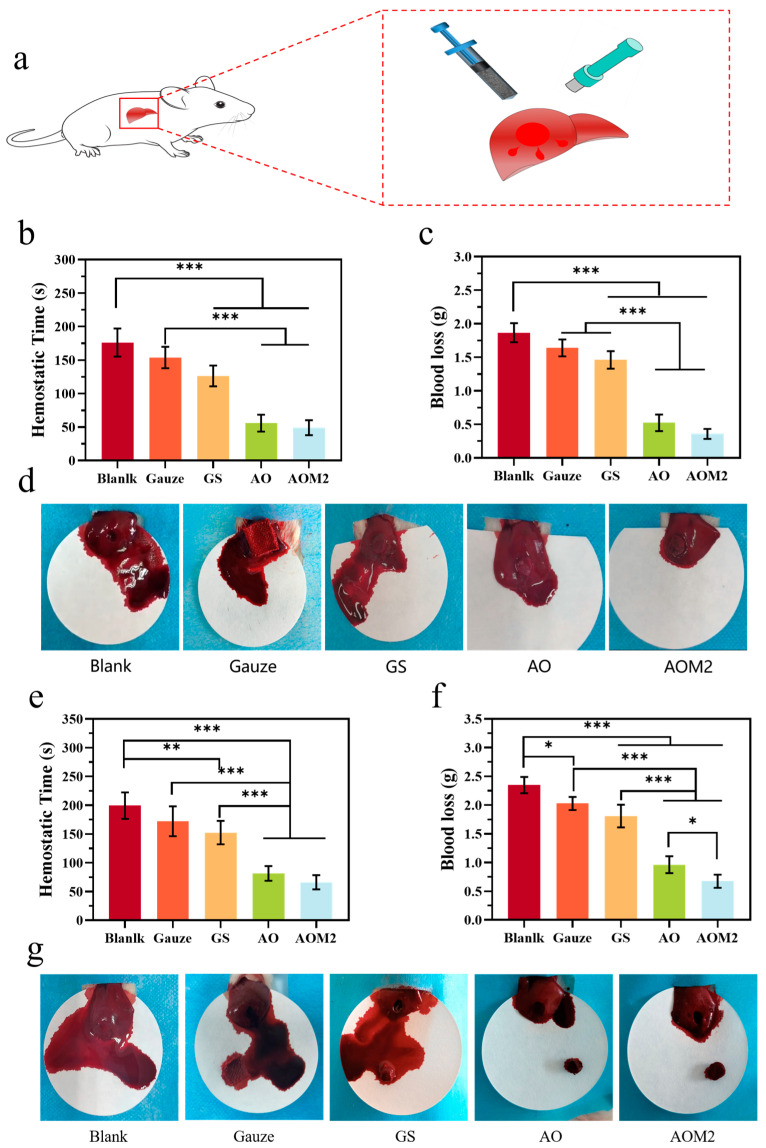
Hemostasis in vivo. (**a**) Model of liver perforation injury in rats; (**b**,**c**) Hemostatic time and blood loss of normal rat liver perforation model; (**d**) Photos of hemostatic effects of gauze, GS, AO and AOM2 in the model of cylindrical liver defect in normal rats; (**e**,**f**) Hemostatic time and blood loss in heparinized rat liver perforation model; (**g**) Photos of hemostatic effect of gauze, GS, AO and AOM2 in heparinized rat liver cylindrical defect model.

Based on the biocompatibility of the cryogel and the BCI experiments, the AOM2 group was chosen for liver hemostasis experiments. AO, GS, gauze, and a blank group were chosen as controls (Figure 8). Bleeding control of anticoagulant patients remains a challenge in clinical settings. The hemostatic effect of the cryogel was evaluated in a liver perforation model in both normal rats and anticoagulated rats with heparinization. Noncompressible wounds were created in the rat’s liver using a 6 mm punch, and the cryogels were injected into the wound site using a syringe, while commercial hemostats were used as indicated for hemostasis (Figure 8a). As illustrated in Figure 8d,g, the AOM2 group exhibited the best hemostatic effect manifested by the smallest area of blood on the filter paper, followed by the AO group. Commercial hemostats demonstrated limited hemostatic efficiency. Figure 8b,c show the quantitative assessment of the hemostatic time and blood loss. The blank group of rats had massive bleeding with a natural hemostasis time of 176.2 ± 8.5 s without external intervention and a blood loss of 1.86 ± 0.13 g. The findings indicated that the commercial hemostats gauze and GS exhibited limited hemostatic effects for noncompressible hemostatic. The blood loss of gauze was 1.64 g ± 0.18 g, followed by that of GS, which was 1.46 ± 0.15 g. The blood loss of cryogels was significantly reduced compared with the control group. The blood loss of AO and AOM2 was 0.52 ± 0.10 g and 0.36 ± 0.16 g, respectively. The hemostasis time of the rats exhibited a similar trend among the groups. The shortest hemostasis time was achieved in the AOM2 group, which was only 49.0 ± 5.8 s, respectively. The findings indicate that both AO and AOM2 are effective hemostats for noncompressible wound bleeding in normal rats, with AOM2 demonstrating the best hemostatic ability.

To assess the hemostatic ability of the hemostatic agents for clotting-impaired, noncompressible wounds, an anticoagulant wound model was developed in rats using heparin. As illustrated in Figure 8a,g, the blank group of heparinized rats bled profusely, with a natural hemostasis time of 199.4 ± 20.25 s and a blood loss of 2.35 ± 0.12 g without external intervention. The blood loss of the gauze group was 2.02 ± 0.21 g, followed by GS with a blood loss of 1.81 ± 0.18 g, demonstrating that commercially available hemostatic agents were less effective in stopping hemorrhage in heparinized wounds, resulting in only about 20% less blood loss compared to the blank group. In contrast, the blood loss in AO and AOM2 was 0.96 ± 0.18 g and 0.67 ± 0.15 g, respectively, representing a reduction of about 65% compared to the blank group. A similar trend was observed in the cryogel group in terms of control of hemostasis time, with AO and AOM2 significantly reducing hemostasis time to 81.4 ± 10 s and 66 ± 8.2 s, respectively, compared to the other groups. AOM2 exhibited greater effectiveness than AO due to its higher mechanical strength, enhanced procoagulant ability, and faster recovery. These findings indicate that the cryogel exhibits a good hemostatic effect on both normal anticoagulated wounds.

### 2.6. Cryogel to Guide Liver Regeneration

The removal of hemostatic material can cause the wound to bleed again in severe wound bleeding. It would be advantageous for patients if the hemostatic agent could be left at the injury site and facilitate tissue regeneration. Thus, the pro-regenerative capacity of AO and AOM2 was examined using their liver regeneration impacts on rats.

**Figure 9:**
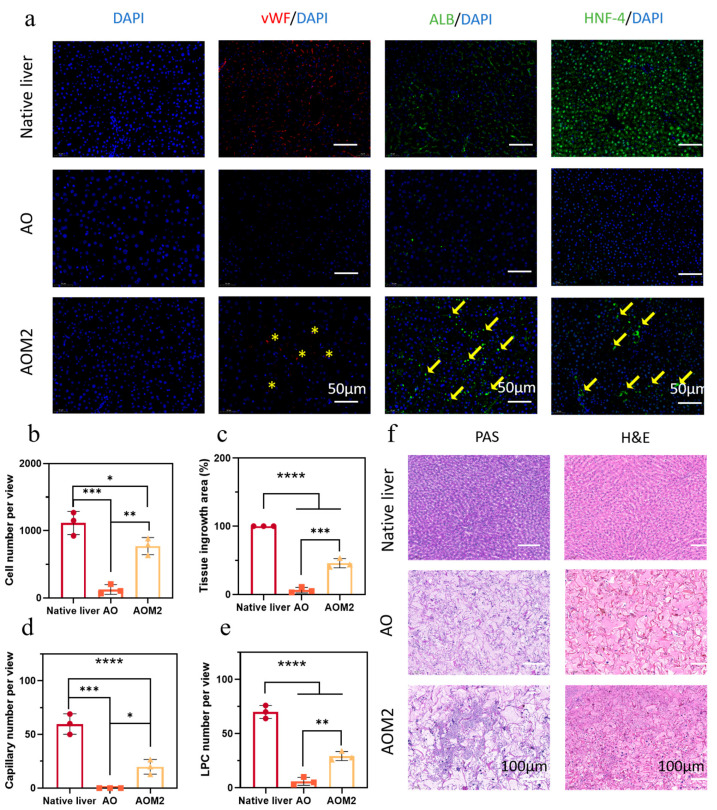
Liver regeneration in rat models after implantation of the AO and AOM2. (**a**) Immunofluorescence staining: DAPI, vWF, ALB, HNF-4; (**b**–**e**) Quantification of cell number, tissue ingrowth area, capillary number, and LPCs within the AO and AOM2; (**f**) PAS staining and H&E staining.

Figure 9 shows the regeneration of the rat liver model after implantation of AO and AOM2. DAPI and H&E staining revealed inward tissue growth (Figure 9a,f), with significantly higher cell numbers in AOM2 than in AO. In Figure 9b, the cells growing into the scaffold formed new tissue. As illustrated in Figure 9f, among other things, the area of tissue growth was significantly larger in AOM2 than in AO, with very little neoplastic tissue observed. The abundance of capillaries is vital for the survival of neoplastic tissue, as they facilitate the delivery of sufficient nutrients and oxygen to the neoplastic tissue, and we evaluated angiogenesis by immunostaining for vascular pseudohemophilic factor (vWF). As demonstrated in Figure 9d, abundant capillaries were distributed in AOM2, unlike in AO where almost no capillary generation was observed. Evaluation of the growth of hepatic parenchymal cells by ALB revealed that more ALB cells grew into AOM2 (Figure 9e), indicating inward growth of hepatic parenchymal cells and regeneration of liver tissue. Minimal growth of hepatic parenchymal cells was also observed in AO. Since HNF-4α is a crucial cell for liver growth, the growth of liver cells was evaluated by detecting HNF-4α. In contrast to AO, where little HNF-4α growth was detected, higher amounts of HNF-4α were observed in AOM2, as demonstrated in Figure 9a. Glycogen, a crucial component of hepatocytes, was observed in AOM2, but it exhibited minimal growth in AO (Figure 9f).

## 3. Conclusions

In this research, we developed a shape-memory AC/ODex/Ag-MBG DFO composite cryogel, which can be employed to control noncompressed bleeding, and exhibits good antibacterial ability, biocompatibility, and tissue regeneration ability. The composite hydrogel achieved an improved hemostatic efficiency through the alkylation of chitosan, the addition of Ag-MBG, and the quick shape recovery of cryogel. In addition, the composite hydrogel effectively promoted hemostasis in anticoagulated animal models, expanding the application scenario of chitosan-based cryogels. Furthermore, the sustained release of proangiogenic agents from the cryogel significantly facilitated liver tissue regeneration, providing the hemostat with wound-healing-promoting functionality. Thus, this organic–inorganic composite cryogel holds great potential for application as a novel multifunctional hemostatic hydrogel.

## 4. Experimental Section

### 4.1. Materials

Shanghai Maclean Biological Co. (Shanghai, China) provided Dex (Dex, Mw = 70,000 Da) and chitosan (CS, Mw = 100,000 Da). Guangzhou Quick Health Medical Devices Co. (Guangzhou, China) provided the gelatin sponge (GS). Sinopharm Holdings Ltd. (Shanghai, China) supplied tetraethyl orthosilicate (TEOS), triethyl phosphate (TEP), silver nitrate (AgNO3), and calcium nitrate tetrahydrate (CN). Beijing Solabao Technology Co. (Beijing, China) provided lysozyme (≥2000 U/mg) and sodium periodate. All other chemical reagents in the text are of analytical grade and can be used directly.

### 4.2. Preparation of Cryogels

AC, ODex, and Ag-MBG were synthesized following the procedures described in the previous literature [20,21,25] (supporting information). The low-temperature gel was prepared using the following steps. First, 0.12 g of alkylated chitosan was added to deionized water, and 60 μL of acetic acid was added to facilitate dissolution. The mixture was stirred until all of the alkylated chitosan was fully dissolved. Second, different concentrations of silver-doped drug-loaded mesoporous bioactive glass were added to the solution from the first step. The mixture was stirred for 30 min to ensure even distribution. Next, 200 µL of 10% (*w*/*v*) ODex was added to the solution while keeping it in an ice bath. The solution was transferred to a refrigerator at −18 °C for 24 h to complete the reaction. Finally, freeze-drying was performed to remove the solvent and obtain the cryogels.

Cryogels containing various concentration gradients of silver-doped drug-loaded mesoporous bioactive glass were prepared using the same method, and the proportions of the constituent components were optimized. Table S1 describes the composition of the formulation of cryogel.

### 4.3. Characterization of Cryogels

The morphology of the silver-doped mesoporous bioactive glass and the surface morphology of the cryogel were examined using scanning electron microscopy (SEM, Carl Zeiss Merlin, Jena, Germany). This analysis aimed to determine the pore size of the cryogel and the particle size of the silver-doped mesoporous bioactive glass. Additionally, the surface morphology of the cryogels was characterized using SEM (Carl Zeiss, Germany), and the obtained SEM images were further analyzed employing ImageJ (ImageJ 1.51) software to measure the pore size of the cryogels. The mesoporous structure of the silver-doped mesoporous bioactive glass was confirmed using transmission electron microscopy (TEM, JEOL, Tokyo, Japan). The specific surface area and pore size distribution of silver-doped mesoporous bioactive glasses were determined using the multipoint N_2_ absorption technique (BET). FT-IR images and 1H NMR images of all materials (CS, AC, Dex, ODex, and Ag-MBG) were recorded using an FT-IR spectrometer and 1H NMR spectrometer (Thermo Scientific Nicolet iS20, Waltham, MA, USA). The solvents employed for characterization were C_2_D_4_O_2_ and D_2_O.

Using elemental analysis, the elemental content ratio of *C* and *N* in the reacted alkylated chitosan (AC) was determined using an elemental analyzer (VARIOEL-III, Germany). The degree of substitution (DS) of AC was computed using the following equation:

$$\frac{C}{N}=\frac{12[6x+8(1-x)+\mathrm{nDS}]}{14}$$

where x represents the degree of deacetylation of CS; n represents the number of *C* atoms on the alkyl chain in AC, taken as 12 in this experiment; DS represents the degree of substitution of the alkyl chain of AC; and *C* and *N* represent the percentage content of carbon and nitrogen elements, respectively.

### 4.4. Characterization of Physical Properties of Cryogels

The physical properties of the cryogels, such as liquid absorption, porosity, mechanical properties, and shape memory properties, were characterized according to previous reports [20]. Details can be found in the supporting information.

### 4.5. Biocompatibility of Cryogels

The biocompatibility of crystalloids was evaluated, and details can be found in the supporting information.

### 4.6. Whole-Blood Coagulation

To determine the whole-blood coagulation of the cryogels, some modifications were made to the reported literature [38]. The in vitro coagulation ability of the cryogels was evaluated using a whole-blood clotting test. The degree of blood coagulation was determined by introducing whole blood into a frozen gel and, at a predetermined time, adding deionized water to lyse uncoagulated RBCs and release hemoglobin. The cryogels were shaped into a cylinder of 5 mm height and 8 mm diameter, dehydrated, and preheated at 37 °C. Subsequently, 100 µL of whole blood (CWB) was dropped into it (10 µL of calcium chloride at 0.2 mol/L was added to the whole blood) and incubated at 37 °C for 5/10 min, shaken for 2 min, and whole blood dissolved in deionized water was employed as a control group. At 540 nm, the absorbance was measured employing a Microplate Absorbance Reader and computed using the following formula:

$$\mathrm{BCI}\;(\%)=\frac{{\mathrm{OD}}_{\mathrm{sample}}}{{\mathrm{OD}}_{\mathrm{reference}\mathrm{value}}}\times 100\%$$

### 4.7. Red Blood Cell and Platelet Adhesion

The adhesion of crystalloids to blood cells and platelets was evaluated, and specific details can be found in the supporting information.

### 4.8. Biodegradability

Cryogel samples were prepared as cylindrical samples with a diameter of 8 mm and a height of 5 mm. Subsequently, the samples were immersed in an excess of lysozyme and allowed to stand at room temperature. The solution was changed every 3 days. The samples were weighed periodically, and the degradation rate was computed for each group. This process was repeated 3 times as follows:

$$\mathrm{Degradation}\mathrm{rate}\;(\%)=\frac{W_{0}-W_{1}}{W_{0}}\times 100\%$$

where W_0_ represents the initial weight of the cryogels and W_1_ represents the weight of the remaining cryogels.

### 4.9. In Vitro Antibacterial Activity

The antibacterial activity of the cryogels was tested against *E. coli* (Gram-negative) and *S. aureus* (Gram-positive). A total of 10 μL of bacterial suspension (105 CFU/mL in PBS) was added to AO, AOM1, AOM2, and AOM3. Then, the 48-well plates were placed in an incubator at 37 °C for 4 h in a relatively humid environment. After the specified time, 990 μL of sterile PBS was added to each well. As a control, 10 μL of bacterial suspension (in PBS, 105 CFU/mL) was added to 990 μL of PBS to obtain a homogenous solution. Colony-forming units (CFU) were counted on the Petri dishes after incubation at 37 °C for 18–24 h. The test was repeated six times for each group, and the kill rate of bacteria by AO, AOM1, AOM2, and AOM3 was measured using the following equation:

$$\mathrm{Bacterial}\mathrm{survival}\mathrm{rate}(\%)=\frac{{\mathrm{OD}}_{\mathrm{test}}-{\mathrm{OD}}_{\mathrm{blank}}}{{\mathrm{OD}}_{\mathrm{control}}-{\mathrm{OD}}_{\mathrm{blank}}}\times 100\%$$

Bactericidal ratio (%) = (1 − Bacterial survival rate (%)) × 100%

where OD_test_, OD_control_, and OD_blank_ represent the OD values of bacterial suspensions containing hydrogel samples, OD values of bacterial suspensions containing LB medium, and OD values of LB medium without bacteria, respectively.

### 4.10. Hemostatic Properties of Cryogels

A test tube containing a 20 mg sample was preheated at 37 °C for 2 min. Then, 2 mL of anticoagulated whole blood was added to the tube, a total of 40 μL of CaCl2 solution (0.2 mol/L) was added to the tube after incubation in a water bath at 37 °C for 3 min. The tubes were tilted every 10 s to observe blood flow. To confirm blood coagulation, the tubes were inverted and observed to see if the blood could rest on its weight. Each set of experiments was repeated three times.

In vivo hemostasis test: The hemostatic performance of AOM2 was assessed by employing a normal/heparin-injected rat-liver perforation-wound model. AO, gauze, and gelatin sponges were employed as controls. The Northwestern University Animal Care and Use Committee (NWU-AWC-20210311R) approved all animal procedures. Normal/heparin-injected rat-liver perforation-wound model: Rats (males, 250–300 g body weight, 7–8 weeks) were anesthetized by injecting a 1 mL/300 g dose of 10 wt% chloral hydrate. Afterward, the rat’s stomach was opened, and the liver was exposed and placed on pre-prepared and weighed filter paper. Circular perforated wounds (6 mm in diameter) were created in the liver to induce bleeding. A prepared cylindrical cryogel (6 mm diameter) was compressed into a syringe and injected into the wound site. The hemostasis process was recorded using a digital camera. The hemostasis time was measured using a timer, and the amount of blood loss was computed by weighing filter paper and the weight of blood absorbed by the hemostatic agent. Anticoagulated rats were modeled by injecting a heparin solution intravenously into the rat’s blood vessels (male, 200–250 g body weight, 2 mL/kg). The blood loss and time to hemostasis were measured using the same method as in the general group. The hemostatic effect of the cryogel on coagulation-impaired noncompressible bleeding was demonstrated.

### 4.11. Regeneration of the Liver

The rat liver regeneration experiment was conducted following a previously documented method [22]. A circular perforated wound with a diameter of 6 mm was created using liver perforation experiments. AO and AOM2 from the experimental group and GS from the control group were filled into the wound. After surgery, the abdomen of the rats was sutured without removing the hemostatic agent, and they were fed normally for 30 days, after which the livers were removed for testing. The infiltration of liver cells was examined using DAPI staining, angiophilic factor (vWF) assessed angiogenesis in liver wounds, albumin (ALB-10)) was used to assess the growth of liver parenchymal cells, hepatocyte nuclear factor (HNF-4α) staining was performed to investigate the expression of liver cytokines, periodate -(PAS) staining was employed to determine whether liver glycogen was synthesized, and H&E staining was conducted to evaluate the growth of liver tissue.

### 4.12. Statistics and Reproducibility

All tests were processed in triplicate and similar results were acquired. Each group has three independent samples. Statistical analyses were performed using GraphPad Prism 8 software. Values are expressed as the means ± standard deviation (SD). Comparison between the two groups was performed by unpaired two-tailed *t*-test. For multiple group comparison, one-way ANOVA with Tukey’s multiple comparison tests was used. * *p* < 0.05 was considered to be statistically significant.

## Data Availability

Data will be available while being asked.

## Supplementary Materials

The following supporting information can be downloaded at: https://www.mdpi.com/article/10.3390/gels9060455/s1, Table S1. Specific composition of different cryogels. Figure S1. (a) Synthesis of ODex. (b) FTIR spectra of Dex and ODex. (c) 1H-NMR spectra of Dex and ODex. Figure S2. (a) Synthesis of alkylated chitosan; (b) FTIR spectra of CS and AC; (c) 1H-NMR spectra of CS and AC. Figure S3. (a) SEM images of the morphology of Ag-MBG; (b) SEM images of the morphology of Ag-MBG. Figure S4. (a) N2 adsorption-desorption isotherm of Ag-MBG; (b) Pore size distribution of Ag-MBG. (c) the XRD spectrum of xx. Figure S5. (a) FTIR spectra of MBG; (b) XRD image of Ag-MBG. Figure S6. FTIR spectra of AOM cryogels. Table S2. Loading of DFO by Ag-MBG. Figure S7. (DFO) drug release profile. Table S3. Effect of ODex content on the properties of cryogels. Table S4. The effect of reaction time on properties of AO cryogel.
