# Supplementary material for: Multifunctional Oxidized Dextran Cross-Linked Alkylated Chitosan/Drug-Loaded and Silver-Doped Mesoporous Bioactive Glass Cryogel for Hemostasis of Noncompressible Wounds
Source: Gels. 2023 Jun 1;9(6):455. doi: 10.3390/gels9060455 (PMC10297613; doi:10.3390/gels9060455)
Supplement: Supplementary file 1 [file gels-09-00455-s001.zip › gels-2366791-supplementary.pdf]

supplementary material

Multifunctional Oxidized Dextran Cross-Linked Alkylated Chitosan/Drug-Loaded and Silver-Doped Mesoporous Bioactive Glass Cryogel for Hemostasis of Noncompressible Wounds

Dong Lei [1,2,3,†], Jing Zhao [1,2,3,†], Chenhui Zhu [1,2,3], Min Jiang [4], Pei Ma [1,2,3], Yu Mi [1,2,3,*] and Daidi Fan [1,2,3,*]

[1] Shaanxi Key Laboratory of Degradable Biomedical Materials, School of Chemical Engineering, Northwest University, Xi’an 710069, China; leidong1010@163.com (D.L.); zhaojing@nwu.edu.cn (J.Z.); zch2005@nwu.edu.cn (C.Z.); mapei@nwu.edu.cn (P.M.)

[2] Shaanxi R&D Center of Biomaterials and Fermentation Engineering, School of Chemical Engineering, Northwest University, Xi’an 710069, China

[3] Biotech & Biomed Research Institute, Northwest University, 229 North Taibai Road, Xi’an 710069, China

[4] State Key Laboratory of Materials-Oriented Chemical Engineering, College of Biotechnology and Pharmaceutical Engineering, Nanjing Tech University, Nanjing 211816, China; jiangmin@njtech.edu.cn

* Correspondence: mi_yu@nwu.edu.cn (Y.M.); fandaidi@nwu.edu.cn (D.F.); Tel.: +86-029-88305118 (Y.M.)

† These authors contributed equally to this work.

1. Experimental Section

1.1. Synthesis of alkylated chitosan

Dissolve 4 g of chitosan (10,000 molecular weight) in 400 ml of deionized water, add 4 ml of acetic acid solution, stir for 1.5 hours at room temperature, then add 2.4 ml of lauric aldehyde to the system, react for 5 h at 35 °C, adjust the pH to 5-5.1 with sodium hydroxide solution (3-5 mol/L); afterwards add 4.8 g of sodium cyanoborohydride to the system, react for 18 h at 45°C The pH was adjusted to 9-10 with sodium hydroxide solution (3-5 mol/L); finally, the alkylated chitosan was obtained by gradient centrifugation (8,000-10,000 rpm, 5-7 min) and freeze-dried at -50°C for 3 days.

1.2. Synthesis of oxidized dextran (ODex)

4 g dextran and 3.4 g sodium periodate were dissolved in 50 mL deionized water and reacted for 24 h in the dark at room temperature. Afterwards, 1 g of ethylene glycol was added to the mixture to terminate the further oxidation of dextran and dialyzed with deionized water for 3 days. Next, the dialyzed solution was freeze-dried to obtain a white sponge-like product (ODex). The oxidation degree of dextran was measured by the hydroxylamine hydrochloride method.

ODex (0.1 g) was dissolved in 20 mL of hydroxylamine hydrochloride-methyl orange solution (hydroxylamine hydrochloride concentration is 0.25 M, 0.05% methyl orange was used as pH indicator) and reacted for 2 h. The mixed solution was then titrated with 0.1 M NaOH and the pH of the solution was recorded.

The oxidation degree of ODex was confirmed using the following formula:

$$\text{Oxidation (\%)} \frac{\Delta V \times 10^{-3} \times M_{NaOH} \times Molecular\ Weight_{Dextran\ monomer}}{0.1} \times 100\%$$

1.3. Synthesis of silver-doped mesoporous bioactive glasses

Place 1.4 g of cetyltrimethylammonium bromide in 66 mL of deionized water and stir for 1h at room temperature to obtain a mixed solution; add 20 mL of ethyl acetate to it,

stir for 30 min, add 14mL of ammonia solution (5-7 mol/L) and stir for 15 h (300-400 rpm); after that add 7.2mL of tetraethyl orthosilicate and continue to stir for 30 min (300-400 rpm), then add 0.72 mL triethyl phosphate, continue stirring for 30 min, add 4.54g calcium nitrate tetrahydrate, continue stirring for 30 min, add 0.4 g silver nitrate, stir vigorously for 4 h (550-600 rpm), centrifuge and obtain a precipitate; said precipitate is white in colour; wash said precipitate with ethanol and water alternately The precipitate is washed 3 times with ethanol and water, dried at 60°C for 24h and calcined in a muffle furnace at 650 °C for 4 hours to remove organic matter and other impurities to obtain silver-doped mesoporous bioactive glass.

1.4(. DFO) loaded into mesoporous bioactive glass

Desferrioxamine and Ag-MBG were immersed in PBS for 48 h at different mass ratios, after which 200 µl of each was removed and mixed with ferric chloride (3 mol/L) and shaken well with ultrasound, after which the OD values were measured at 482 nm under UV spectrophotometer. The loading and encapsulation rates of the drugs were obtained by bringing the standard curve.

$$\text{Drug loading rate (\%)} = \frac{\text{weight of drug in the carrier}}{\text{weight of carrier}} \times 100\%$$

$$\text{Encapsulation rate (\%)} = \frac{\text{weight of drug in the carrier}}{\text{total drug}} \times 100\%$$

1.5. Release of drugs

0.1 g of drug-loaded Ag-MBG was added to 10 ml of PBS and three parallel sets of experiments were performed. 200 µl of supernatant was pipetted out every 24 h, while 200 µl of PBS was added to the system for 10 days, after which the supernatant was homogeneously mixed with ferric chloride (3 mol/L) at a volume ratio of 1:1 and the OD value was measured at 482 nm under a UV spectrophotometer and substituted into the The standard curve was used to obtain the drug release rate.

$$\text{Drug release rate (\%)} = \frac{\text{weight of drug in supernatant}}{\text{drug load}} \times 100\%$$

1.6. Characterisation of the physical properties of cryogels

The uptake rate of the cryogels was measured in PBS/blood. The liquid uptake rate was calculated as follows:

$$\text{PBS absorptivity (\%)} = \frac{(W_1 - W_0)}{W_0} \times 100\%$$

Where W_1 refers to the weight of the cryogel removed from the liquid and W_0 refers to the weight of the freeze-dried cryogel.

The porosity of the cryogel is calculated by means of liquid displacement and is calculated as follows:

$$\text{Porosity (\%)} = \frac{(M_1 - M_0)}{\rho V} \times 100\%$$

where M_0 refers to the weight of the freeze-dried cryogel, M_1 refers to the weight of the cryogel removed from the ethanol, ρ refers to the density of the ethanol (0.789 g/cm^3) and V refers to the volume of the cryogel

For the mechanical properties tests, a universal compressor was used with a compression rate of 2 mm/min and a maximum strain of 80%.

The shape memory properties of the cryogel were calculated using the following equation:

$$\text{Shape recovery rate (\%)} = \frac{H_1}{H_0} \times 100\%$$

where H_0 refers to the initial height of the cryogel and H_1 refers to its height after it has recovered its shape.

The shape memory properties of the cryogels were investigated according to literature[12]. The cryogels were compressed to 80% of their initial height using a universal testing machine (INSTRON 5565, USA), then some of the compressed samples were immersed in PBS/blood and the time taken to recover the shape and the length after recovery were recorded. Afterwards, the cryogels were cut longitudinally, sprayed with gold and the original, compressed and recovered states of the cryogels were observed using scanning electron microscopy (SEM).

1.7. Biocompatibility of cryogels

The biocompatibility of the cryogels, including Hemocompatibility and cytocompatibility, is assessed by haemolysis tests and cytotoxicity tests respectively.

Hemocompatibility: The RBC was diluted to a 5% RBC suspension with PBS. The cryogels were pulverized into powder with a crusher and then dispersed in PBS to make a suspension of 10 mg/mL concentration.

After pre-warming the suspension at 37 °C for 30 minutes, 0.5 mL of 5% RBC suspension was added to the sample suspension (0.5 mL), incubated at 37 °C for 1 h, and then centrifuged at 1500 rpm for 10 minutes.

The absorbance of the supernatant was measured at 540 nm (OD_{sample}). 0.5 mL of 5% RBC suspension was added to 0.5 mL of 0.1% Triton X-100, and PBS served as positive and negative controls, respectively. The hemolysis rate was calculated by the following equation:

$$Hemolysis\ ratio\ (\%) = \frac{OD_{sample} - OD_{negative\ control}}{OD_{positive\ control} - OD_{negative\ control}} \times 100\%$$

Cytocompatibility: The cytocompatibility of the cryogels was evaluated by MTT and AO/EB staining. Mouse fibroblasts (L929) as the cell line were maintained at 37 °C in 5% CO_2 in RPMI-1640 medium supplemented with 10 % fetal bovine serum (FBS) and 1% penicillin-streptomycin.

Briefly, the cryogels sterilized by Co60 were immersed in 1640 medium at 37 °C for 24 h to obtain an extract of 10 mg/mL. L929 cells (1000 cells per well) were inoculated into 96-well plates. After 24 h incubation, the culture medium was replaced with the cryogels extracts (100 μL/well) and incubated in the cell incubator for 24, 48, and 72 h before 50 μL of MTT solution was added to each well and incubated for 2 h. Then the solution in each well was removed, and 150 μL DMSO was added. Finally, the absorbance of the solution was measured at 490 nm (OD_{sample}). A negative control (without extract) was included at each time point ($OD_{negative\ control}$).

The cell viability was calculated by the following equation:

$$\text{Cell viability (\%)} = \frac{OD_{sample}}{OD_{negative\ control}} \times 100\%$$

Meanwhile, to observe the morphology and survival of L929 cells, the extracts were added, and after incubation for 24 h, the AO/EB staining kit (Leigen, Beijing, China) was used for staining. Finally, the cells were observed by an inverted fluorescence microscope (Olympus, Japan).

1.8. Red blood cell and platelet adhesion

The interaction between the cryogels and RBCs was studied using the previously reported method with some modifications. Gauze, gelatin sponges, and AC/ODEX were used as controls. Prior to testing, suspensions of RBCs were obtained by centrifuging CWB at 400 x g for 10 minutes. The cryogel was compressed to drain water and placed in a 24-

well microtiter plate. Next, 100 μL of erythrocyte suspension was dropped onto their top surface. After incubation at 37°C for 1 hour, the suspension was rinsed with phosphate buffer (PBS, pH = 7.4) to remove unadhered RBCs and then transferred to DIW (4 mL) to lyse adhered RBCs to release haemoglobin. 1 hour later, 100 μL of supernatant was removed and placed in a 96-well microtiter plate and its OD 540 nm value was measured. The OD 540 nm value of a solution consisting of 100 μL of erythrocyte suspension and 4 mL of DIW was used as a reference value ($OD_{\text{reference value}}$). The percentage of adherent red blood cells was calculated by the following formula:

$$\text{RBC adhesion (\%)} = \frac{OD_{\text{hemostat}}}{OD_{\text{reference value}}} \times 100\%$$

The interaction between the various hemostatic agents and platelets was further assessed by platelet adhesion assays. Platelet-rich plasma (PRP) was obtained by centrifuging CWB at 400 x g for 10 minutes prior to measurement. The cryogel was compressed to squeeze out water and placed in a 24-well microtiter plate. Then, 100 μL of PRP was dropped onto their upper surface and subsequently incubated at 37°C for 1 hour. Next, they were washed with PBS to remove unadhered platelets and immersed in 1% Triton X-100 solution to lyse the platelets and release lactate dehydrogenase (LDH). LDH was measured with an LDH kit (Beyoncé, China) according to its instructions. Finally, the OD 490 nm value of the supernatant was measured and called the OD haemostat. The OD 490 nm value of a solution consisting of 100 μL of unexposed PRP and hemostatic agent was measured and used as a reference value ($OD_{\text{reference value}}$). The percentage of adherent platelets was calculated by the following equation:

$$\text{Adhered platelet (\%)} = \frac{OD_{\text{hemostat}}}{OD_{\text{reference value}}} \times 100\%$$

Scanning electron microscopy was used to observe the adhesion of erythrocytes and platelets to different hemostatic agents. Briefly, hemostatic agents were placed into each well of a 24-well microtiter plate and contacted with 100 μL of erythrocyte and PRP suspension. after 1 hour at 37°C, they were rinsed with PBS, then fixed with 2.5% glutaraldehyde and dehydrated with a series of graded alcohol solutions. After drying, they were cut and sectioned longitudinally with gold sputtering and observed with SEM. Activated platelets adhered to gauze, gelatin sponges, AC/ODex and AC/ODex/Ag-MBG surfaces.

1.9. Collection and preparation of blood-related components

Blood-related component collection and preparation Citrate whole blood (CWB) was extracted from healthy male New Zealand White Rabbits using a sodium citrate anticoagulant tube. The ratio of blood to 3.8% w/v sodium citrate was 1:9.

CWB was centrifuged at 1500 rpm for 10 minutes, and the supernatant was platelet-rich plasma (PRP). The precipitate was washed with PBS three times to obtain red blood cell suspension (RBC).

CWB was centrifuged at 3000 rpm for 15 minutes, and the supernatant was platelet-poor plasma (PPP).

2. Supporting Results

Table S1. Specific composition of different cryogels.

Cryogel	AC (%，w/v)	ODex (%，w/v)	Ag-MBG DFO(%，w/v)
AO	1.2	0.5	0
AOM1	1.2	0.5	0.2
AOM2	1.2	0.5	0.4
AOM3	1.2	0.5	0.6

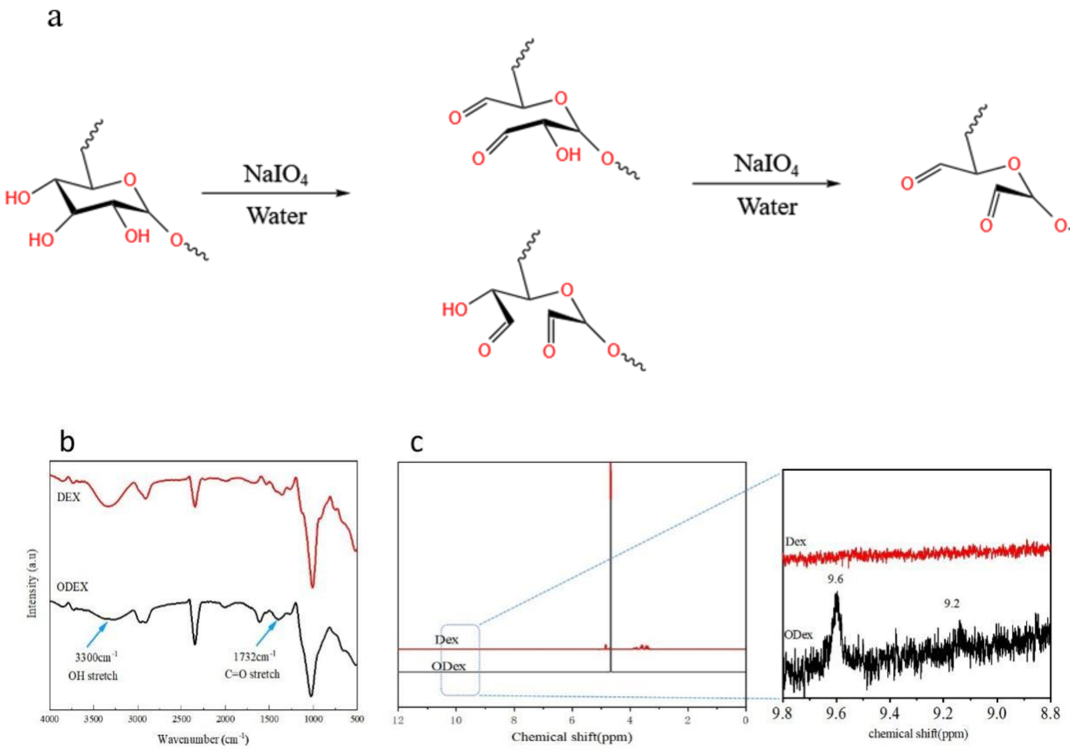

Figure.S1. (a) Synthesis of ODex. (b) FTIR spectra of Dex and ODex. (c) 1H-NMR spectra of Dex and ODex.

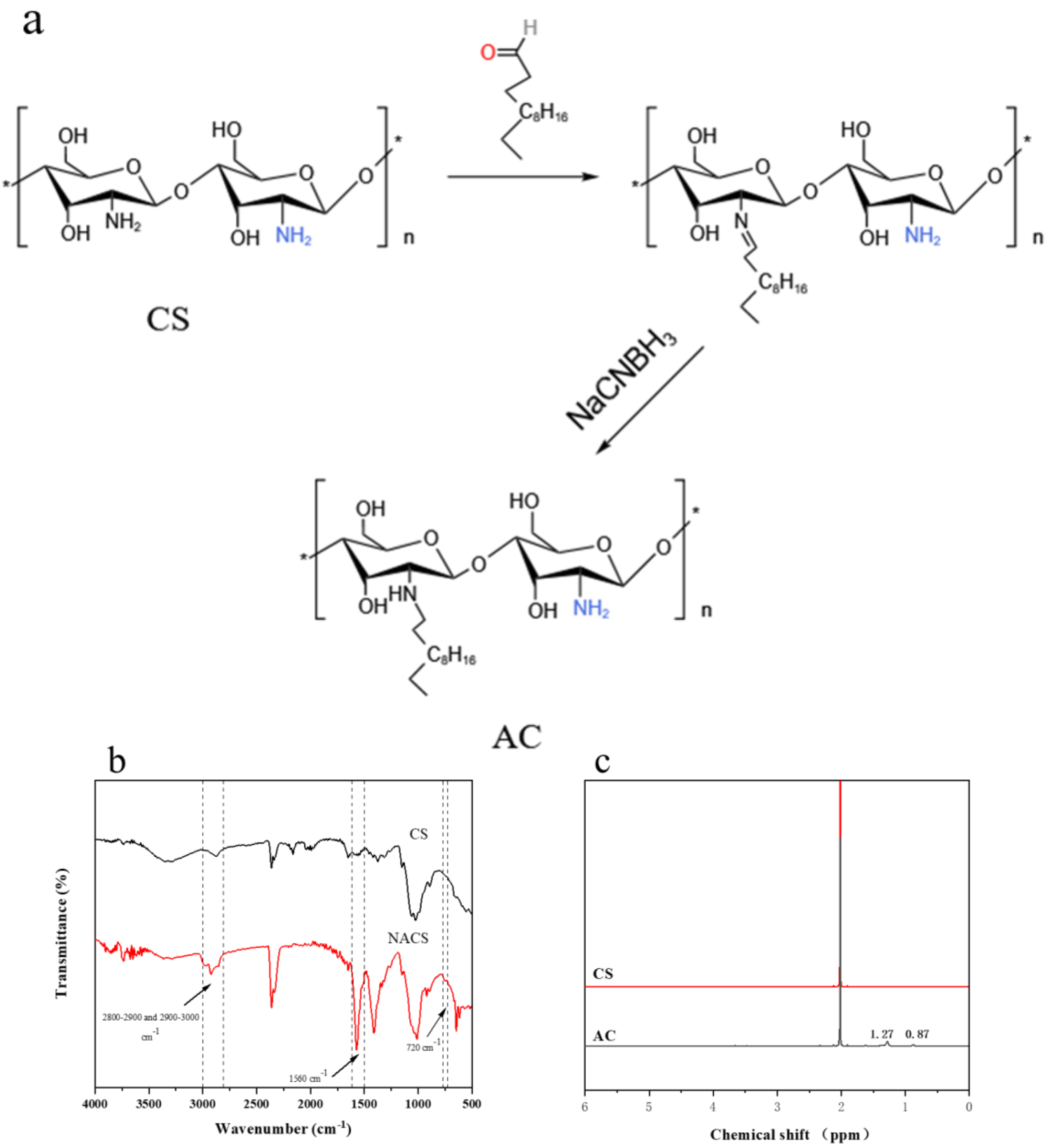

Figure S2. (a) Synthesis of alkylated chitosan; (b) FTIR spectra of CS and AC; (c) 1H-NMR spectra of CS and AC.

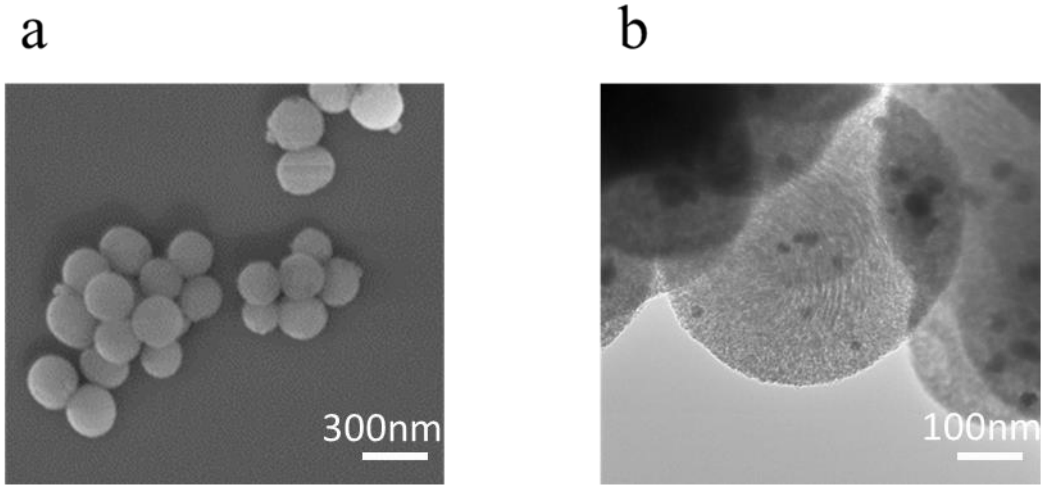

Figure S3. (a) SEM images of the morphology of Ag-MBG; (b) SEM images of the morphology of Ag-MBG. The scale bars in (a): 300 nm, (b):100nm.

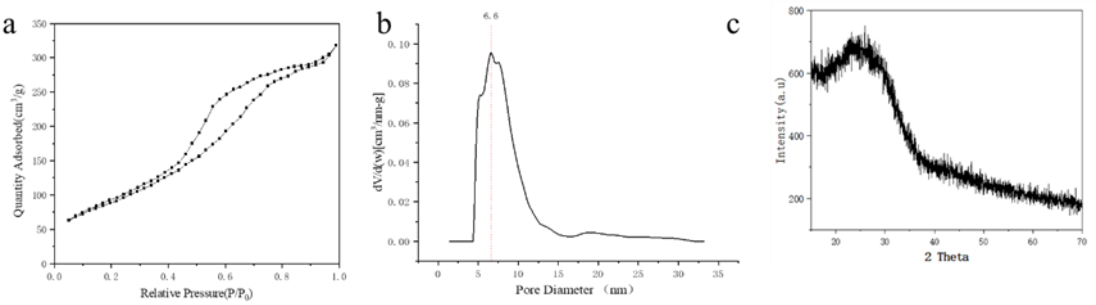

Figure S4. (a) N_2 adsorption-desorption isotherm of Ag-MBG; (b) Pore size distribution of Ag-MBG. (c) the XRD spectrum of xx.

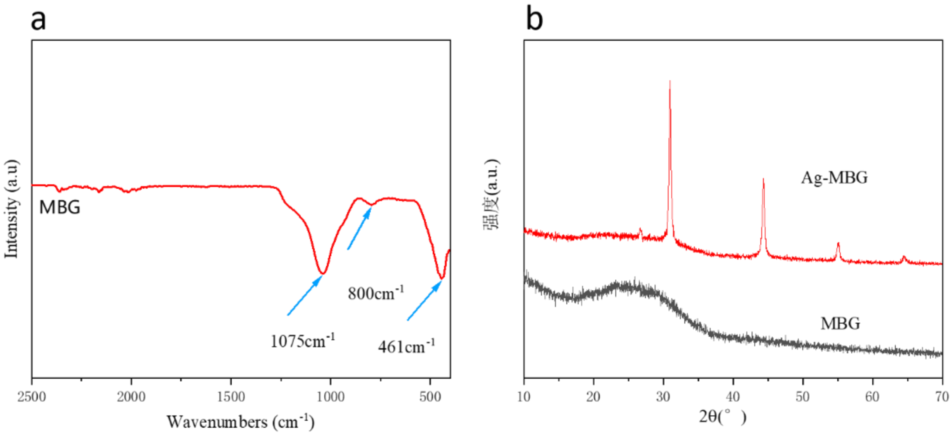

Figure S5. (a) FTIR spectra of MBG; (b) XRD image of Ag-MBG.

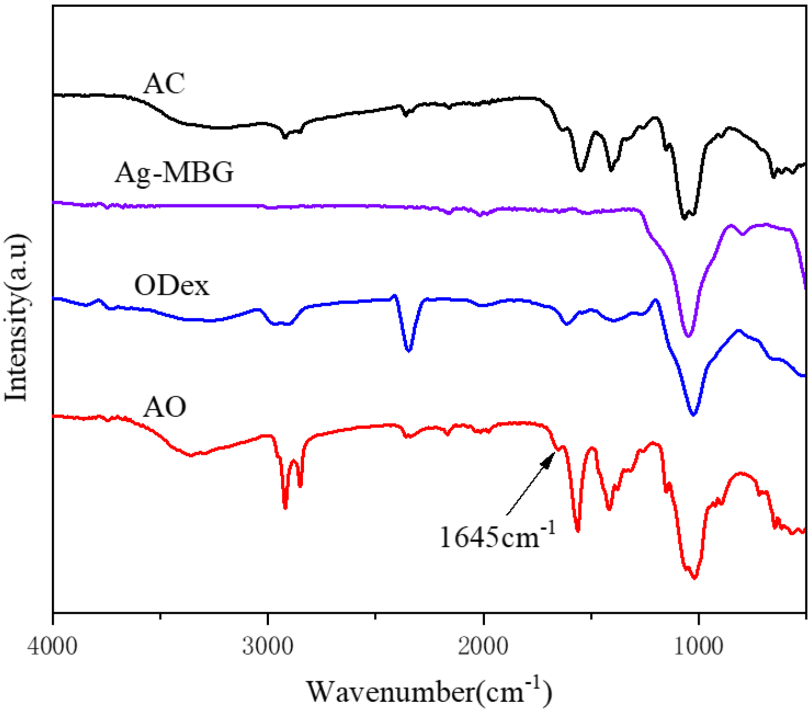

Figure S6. FTIR spectra of AOM cryogels.

Table S2. Loading of DFO by Ag-MBG.

Ag-MBG:DFO (Mass ratio)	10:1	10:3	10:6	10:9	10:12	10:15
DFO (mg)	1	3	6	9	12	15
Drug loading rate (%)	4.93	7.33	15.19	22.42	50.88	42.37

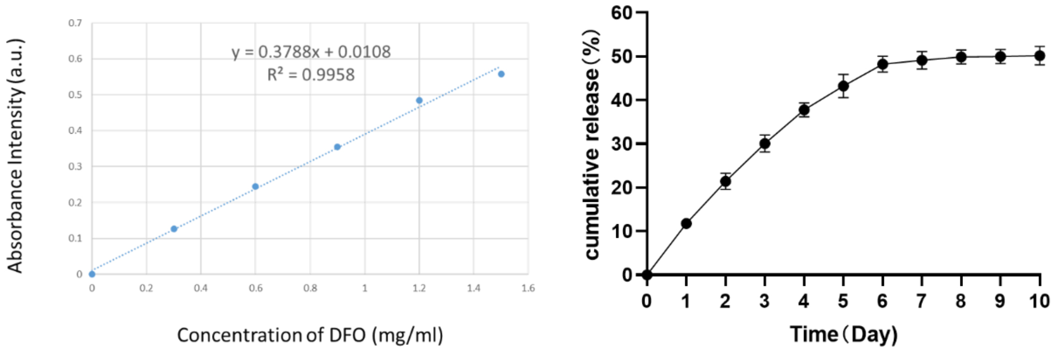

Figure S7. (DFO) drug release profile.

The ideal cryogels should have a stable shape memory, excellent coagulation properties and good liquid absorption. rate. Since Ag-MBG DFO is introduced into the system by physical adhesion to the crystal gel surface, it does not participate in chemical bonding.

Therefore, in order to investigate the effect of the appropriate ODex concentration on the properties of the prepared cryogels, the optimal concentration of the cryogels was investigated in a single-factor experiment using the shape memory capacity, coagulation index and absorption rate of PBS of AO cryogels as evaluation indicators.

Table S3. Effect of ODex content on the properties of cryogels.

ODex(%,w/v)	Shap recovery time(S)	Coagulation index	PBS absorption rate
0.5	5.3	27.1	5050.3
0.75	7.2	28.5	4765.4
1	9.6	31.2	4255.8

Table S4. The effect of reaction time on properties of AO cryogel.

Times(h)	Shap recovery time(S)	Coagulation index	PBS absorption rate
18	7.6	32.5	4896.2
24	5.2	28.8	5018.7
36	5.1	27.9	5115.5

Finally, we explored the effect of reaction temperature on cryogel formation. The reaction temperature was set to be -20 °C, -40 °C and -80 °C, and it was found that the cryogel could not be formed at -40 °C and -80 °C. The possible reason is that the reaction temperature is too low and the movement between molecules is too slow, resulting in an incomplete reaction with very low cross-linking density, which eventually leads to the failure of the reaction. Therefore, the reaction temperature of cryogels was set at -20 ℃ in this study. From the experimental results (Table S3 and Table S4), it can be seen that the optimal conditions for the synthesis of AO cryogels are: reaction temperature of -20 ℃, reaction time of 24h, and the concentration of ODex added is 0.5% (w/v).
